# Screening for characteristic microRNAs between pre-invasive and invasive stages of cervical cancer

**DOI:** 10.3892/mmr.2015.3363

**Published:** 2015-02-17

**Authors:** XIAO-LU ZHU, SHANG-YUN WEN, ZHI-HONG AI, JUAN WANG, YAN-LI XU, YIN-CHENG TENG

**Affiliations:** Department of Obstetrics and Gynecology, Shanghai Sixth People’s Hospital, Shanghai Jiaotong University, Shanghai 200233, P.R. China

**Keywords:** cervical cancer, invasion, differentially expressed gene, gene ontology, microRNA-target gene network

## Abstract

The aim of the present study was to investigate the characteristic microRNAs (miRNAs) expressed during the pre-invasive and invasive stages of cervical cancer. A gene expression profile (GSE7803) containing 21 invasive squamous cell cervical carcinoma samples, 10 normal squamous cervical epithelium samples and seven high-grade squamous intraepithelial cervical lesion samples, was obtained from the Gene Expression Omnibus. Differentially expressed genes (DEGs) were identified using significance analysis of microarray software, and a Gene Ontology (GO) enrichment analysis was conducted using the Database for Annotation, Visualization and Integrated Discovery. The miRNAs that interacted with the identified DEGs were selected, based on the TarBase v5.0 database. Regulatory networks were constructed from these selected miRNAs along with their corresponding target genes among the DEGs. The regulatory networks were visualized using Cytoscape. A total of 1,160 and 756 DEGs were identified in the pre-invasive and invasive stages of cervical cancer, respectively. The results of the GO enrichment demonstrated that the DEGs were predominantly involved in the immune response and the cell cycle, in the pre-invasive and invasive stages, respectively. Furthermore, a total of 18 and 26 characteristic miRNAs were screened in the pre-invasive and invasive stages, respectively. These miRNAs may be potential biomarkers and targets for the diagnosis and treatment of the different stages of cervical cancer.

## Introduction

Cervical cancer is the most common gynecological malignancy, and is the second leading cause of cancer-associated mortality in females worldwide (1,2). One reason for the high levels of prevalence of this cancer is the lack of awareness and early detection approaches (3,4). Therefore, understanding the underlying molecular mechanisms of cervical cancer, and establishing more effective therapies are important areas of ongoing research.

The identification and characterization of key microRNAs (miRNAs) that participate in cervical cancer, is essential for determining the underlying mechanisms of this disease and establishing novel therapeutic strategies. miRNAs are 20–24 nt RNAs that are derived from distinct hairpin precursors in animals, plants and fungi, which bind to complementary sequences on target mRNAs (5,6). miRNAs regulate gene expression by cleaving target mRNAs, and by translational suppression at the post-transcriptional level (7). Previous studies have shown that miRNAs have important roles in various biological and metabolic processes, including cell growth, apoptosis, viral infection, differentiation, signal transduction and cancer (8–11). Numerous studies have demonstrated that miRNAs are involved in the initiation and progression of cancer, and may be potential biomarkers for the diagnosis and prognosis of tumors, in addition to functioning as potential therapeutic targets (12–14). Therefore, it may be beneficial to identify novel miRNAs to act as diagnostic and therapeutic biomarkers, or therapeutic targets, in cervical cancer.

Recently, molecular network analysis technology, combined with gene expression profile data, has exhibited potential in a number of areas, including classification of diseases and the identification of novel therapeutic targets (15,16). In the present study a microarray dataset of healthy and malignant cervical samples was downloaded from the Gene Expression Omnibus (GEO) database. Differentially expressed genes (DEGs) were identified between these groups. Based on the TarBase v5.0 database, regulatory networks were constructed from selected miRNAs and their corresponding target genes from the identified DEGs. Key miRNAs, which may be used as potential biomarkers or therapeutic targets in cervical cancer, were subsequently identified.

## Materials and methods

### Affymetrix microarray data

A gene expression profile generated by Zhai *et al* (17) was used in the present study, which was deposited in the GEO database (http://www.ncbi.nlm.nih.gov/geo/query/acc.cgi?acc=GSE7803). This gene expression profile is based on the GPL96 platform (Affymetrix Human Genome U133A Array). A total of 38 samples were available, including 21 invasive squamous cell cervical carcinoma (SCC) samples, ten normal squamous cervical epithelium (NE) samples and seven high-grade squamous intraepithelial cervical lesion (HSIL) samples.

### Screening of DEGs

In order to identify the DEGs, the original GSE7803 dataset was converted into an identifiable expression form and was normalized. Probe sets were mapped to the National Centers of Biotechnology Information genes (http://www.ncbi.nlm.nih.gov). Probe sets that corresponded to numerous genes or to no genes were removed from subsequent analyses. For genes that corresponded with numerous probe sets and had a plurality of expression values, the expression values were averaged. Subsequently, the SAMR package (18) in R and a significance analysis of microarray (SAM) were used to identify the DEGs between the samples (19). SAM software is a practical tool used for detecting significantly expressed genes, and for controlling the proportion of falsely detected genes. In the present study, genes with a fold-change >1.2 and a false discovery rate (FDR) <0.05 were selected as DEGs. In addition, the identified DEGs were divided into two groups: DEGs from the NE and HSIL samples were considered pre-invasive DEGs, whereas DEGs from the HSIL and invasive SCC samples were considered invasive DEGs.

### Functional enrichment analysis of DEGs

The Database for Annotation, Visualization and Integrated Discovery (DAVID; http://david.abcc.ncifcrf.gov/) is a web-accessible program that provides a comprehensive set of functional annotation tools, which may be used by investigators to understand the underlying biological functions of large lists of genes (20). The present study used DAVID to perform a Gene Ontology (GO) enrichment analysis of the identified DEGs. Based on hypergeometric distribution, GO terms were enriched, and numerous testing corrections were conducted using the Benjamini-Hochberg method (21). An FDR<0.05 was set as the cut-off value.

### Construction of regulatory networks

TarBase is a database that contains a manually curated collection of experimentally supported miRNA targets from a animal, pant and viral species of central scientific interest (22). TarBase v5.0 is the updated and extended version of the TarBase database, with >1,300 experimentally supported miRNA-target interactions (MTIs). It contains 1,094 human MTIs between 285 miRNAs and 1,721 target genes.

In the present study, human miRNA target gene data were downloaded from the TarBase v5.0 database (http://diana.cslab.ece.ntua.gr/tarbase/). miRNAs that interacted with the identified DEGs were then selected. Subsequently, MTIs regulatory networks were constructed from these selected miRNAs and their corresponding target genes within the DEGs. The MTIs regulatory networks were visualized by Cytoscape (23). In addition, the MTIs regulatory networks were divided into two groups: The regulatory network constructed from the selected miRNAs and the pre-invasive DEGs was termed the pre-invasive regulatory network, whereas the regulatory network constructed from the selected miRNAs and the invasive DEGs was termed the invasive regulatory network.

### Comparison of the regulatory networks

In order to determine the differences between the pre-invasive and invasive stages of cervical cancer, regulatory networks were constructed and compared. Regulatory networks may be characterized by topological properties, such as degree (24). Degree is defined as the number of edges per node, which indicates the number of interacting partners. The present study used Freeman’s degree centrality to analyze the degree of the regulatory networks (25). Freeman’s degree centrality consists of ingoing (in-degree) and outgoing degree (out-degree). In-degree refers to the number of links a node receives from other nodes, whereas out-degree refers to the number of links originating from a particular node.

## Results

### DEG analysis

The original GSE7803 dataset was downloaded from the GEO database, and the DEGs were identified using SAM. Genes with a fold-change >1.2 and an FDR <0.05 were classed as DEGs. A total of 1,160 pre-invasive and 756 invasive DEGs were identified. In addition, 2,001 DEGs were identified from the NE and invasive SCC samples.

### GO analysis of DEGs

In order to study the DEGs that contributed to cervical cancer, a GO enrichment analysis for the pre-invasive and invasive DEGs was performed using DAVID software. The pre-invasive DEGs (e.g. PSMB10, POU2AF1, ST6GAL1, CLU, SERPING1 and APOL2) were predominantly involved in the immune response, such as the acute inflammatory response (FDR=3.06E-04; Table I). By contrast, the invasive DEGs (e.g. TTK, AURKA, BRCA2, PSMC3IP, CDK10 and TUBG1) were predominantly involved in the regulation of the cell cycle, such as Cell Cycle (FDR=1.25E-19; Table II).

### Construction of regulatory networks

**Figure 1:**
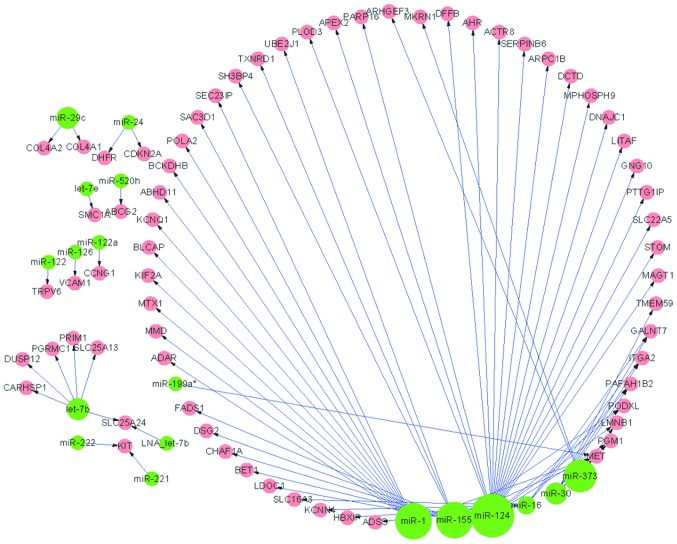
Pre-invasive regulatory network. Regulatory network constructed by microRNAs and differentially expressed genes from normal squamous cervical epitheilum and high grade squamous intraepithelial cervical lesion samples. Red nodes represent target differentially expressed genes and green nodes represent microRNAs. Blue lines represent microRNA-target regulatory interactions (in-degree), and arrows indicate microRNA target differentially expressed genes (out-degree). The size of each green node represents the out-degree. As the out-degree increases, the associated green node becomes larger.

**Figure 2:**
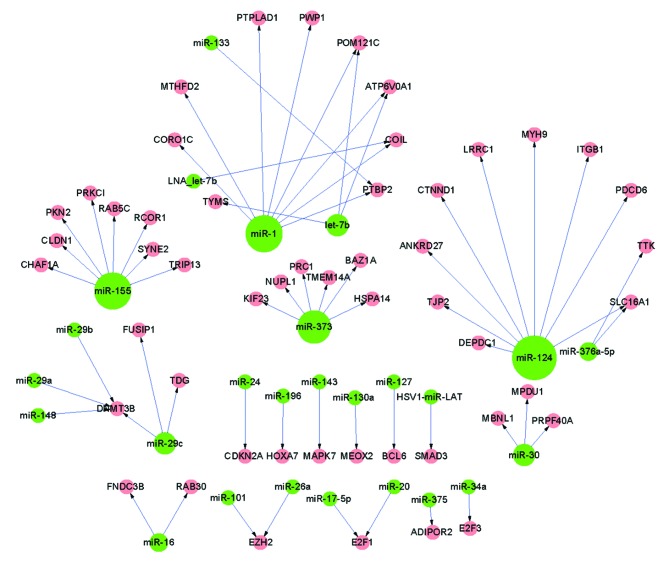
Invasive regulatory network. Regulatory network constructed by microRNAs and differentially expressed genes from invasive squamous cell cervical carcinoma samples and high grade squamous intraepithelial cervical lesion samples. Red nodes represent target differentially expressed genes and green nodes represent microRNAs. Blue lines represent microRNA-target regulatory interactions (in-degree), and arrows indicate microRNA target differentially expressed genes (out-degree). The size of each green node represents the out-degree. As the out-degree increases, the green node becomes bigger.

Based on human MTIs data, pre-invasive and invasive regulatory networks were constructed. The pre-invasive regulatory network consisted of 80 pairs of regulatory interactions between 18 miRNAs and 66 pre-invasive DEGs (Fig. 1). The invasive regulatory network consisted of 64 pairs of regulatory interactions between 26 miRNAs and 51 invasive DEGs (Fig. 2). The highest out-degree was observed in miR-124, in the pre-invasive as well as the invasive regulatory networks.

### Comparisons between the regulatory networks

Based on the topological properties of the networks, the similarities and differences between the pre-invasive and invasive regulatory networks were identified. The invasive regulatory network (Fig. 2) consisted of many smaller sub-networks and the out-degree of miRNAs was decreased, compared with those in the pre-invasive regulatory network (Fig. 1). For example, there were 14 DEGs associated with miR-1, and 21 DEGs associated with miR-124 in the pre-invasive regulatory network (Fig. 1). However, only eight and nine DEGs were associated with miR-1 and miR-124 in the invasive regulatory network, respectively (Fig. 2).

A total of 10 common miRNAs were identified in the regulatory networks. Three miRNAs: miR-1, miR-124 and miR-16, had a degree change >5. In addition, there were eight miRNAs that were only detected in the pre-invasive regulatory network (Fig. 1), including miR-126 and miR-199a. By contrast, there were 16 miRNAs that were only detected in the invasive regulatory network (Fig. 2), including miR-127, miR-143, miR-17-5p, miR-26a, miR-29a, miR-34a and miR-375.

## Discussion

Malignant transformation during tumor progression results from a series of genetic alterations (26). In order to gain a better understanding of the genetic changes that occur during the progression of cervical cancer, a gene expression profile (GSE7803) was analyzed using a bioinformatics approach. In the present study, a total of 756 invasive DEGs, 1,160 pre-invasive DEGs, and 2,001 DEGs from invasive SCC and NE samples, were identified. These findings are in accordance with those of previous studies, which have consistently shown that the expression of genes is markedly altered in invasive tumor cells, compared with that of noninvasive and normal cells (27,28). Furthermore, the results of a GO enrichment of the identified DEGs, indicated that the expression of key genes differs between the pre-invasive and invasive stages of cervical cancer.

Clusterin (CLU) was initially identified as a secreted glycoprotein that has a cytoprotective role. However, numerous intracellular CLU variants have recently been identified in diverse pathological conditions (29–31). Furthermore, recent studies have shown that CLU is involved in various biological functions, such as cell death, tumor progression and neuro-degenerative disorders (32,33). A previous study used DNA microarray data to identify novel candidate molecular markers for cervical cancer diagnosis and therapy, and observed the downregulation of human C1 inhibitor (SERPING1) in invasive cervical carcinoma cells (34). In addition, a recent genomic study demonstrated that apolipoprotein L2 (APOL2) is markedly upregulated in cervical cancer (35). These findings, as well as the results of the present study, indicate that CLU, SERPING1 and APOL2 may have important roles in the progression of cervical cancer.

TTK has been shown to be associated with metastasis via chromosomal instability, in a previous study, which aimed to identify genes associated with the progression and metastasis of advanced cervical cancer following radiotherapy (36). Furthermore, genetic variants of Aurora A kinase (AURKA) have been shown to be associated with a radiotherapy-induced early adverse reaction in patients with cervical cancer (37). Previous studies have demonstrated that both BRCA1 and BRCA2 participate in a common DNA damage response pathway, and are involved in the activation of homologous recombination and double-strand break repair (38). By contrast, Narayan *et al* (39) reported the downregulation of BRCA1 in a small subset of patients with cervical cancer. These previous findings and the results of the present analysis suggest that TTK, AURKA and BRCA2 may participate in the progression of cervical cancer.

In order to obtain the upstream regulatory information of the DEGs, two regulatory networks were constructed based on the TarBase v5.0 database. These regulatory networks were then compared, and the common and specific miRNAs were identified. The miRNA with the highest out-degree was shown to be miR-124, in the pre-invasive as well as the invasive regulatory networks. miR-124 has previously been shown to be the most abundant miRNA expressed in neuronal cells (40). Furthermore, previous studies have shown that the upregulation of miR-124 induces neuronal differentiation of various tumor cell lines in mice (41-43). Wilting *et al* (44) previously demonstrated that the silencing of miR-124 expression, by methylation, inhibited the development of cervical carcinoma. These results suggest that miR-124 may be a potential therapeutic target for cervical cancer therapy.

Of the eight miRNAs specific to the pre-invasive regulatory network, miR-126 has previously been reported to be downregulated in cervical cancer tissues (45), and miR-199a has previously been suggested as a potential therapeutic target for cervical cancer therapy (46). miR-126 is a human miRNA that is expressed only in endothelial cells, throughout capillaries as well as in larger blood vessels (47), and acts upon various transcripts in order to control angiogenesis (48). miR-126 has been identified as a tumor suppressor and as an oncogene, depending on the type of cancer involved. Inhibition of cancer progression by miR-126 is achieved through the negative control of proliferation, migration, invasion and cell survival. However, miR-126 may also support cancer progression through the promotion of blood vessel formation and inflammation at the site of activation (49). According to these previous findings and the results of the present study, miR-126 may be a potential biomarker for the diagnosis of cervical cancer, and a therapeutic target for the pre-invasive stage of this disease.

Of the 16 miRNAs specific to the invasive regulatory network, seven have been reported in previous studies and described as being upregulated or downregulated in cervical cancer. These include miR-127, miR-143, miR-17-5p, miR-26a, miR-29a, miR-34a and miR-375 (45,50–54). Lee *et al* (46) demonstrated that the expression of miR-127 was significantly increased in patients with invasive squamous cell carcinoma, which had metastasized to the lymph nodes. The results of the present study are in accordance with those of previous studies, which indicate that miR-127 may be a marker for lymph node metastasis in invasive cervical cancer. miR-143 is highly conserved in vertebrates (55) and changes in miR-143 expression have frequently been implicated in cancer (56–58). Furthermore, the upregulation of miR-143 has previously been observed in a hepatocellular carcinoma model during tumor metastasis, through repression of FNDC38 (59). However, reduced expression of miR-143 has also been observed in a range of cancer stages, including at very early stages (60). The results of previous studies and of the present study indicate that miR-143 may be involved in tumor progression, and may be a candidate for RNA-targeted treatment of tumors (61). Wang *et al* (52) previously reported that miR-375 is downregulated in squamous cervical cancer, and inhibits cell migration and invasion by targeting the transcription factor, SP1. This finding indicates that deregulation of miR-375 may have an important role in the malignant transformation of cervical cancer cells. However, the elucidation of the underlying molecular mechanisms of miR-17-5p, miR-26a, miR-29a and miR-34a in the progression of cervical cancer, and the use of other miRNAs screened in the present study as biomarkers or therapeutic targets in cervical cancer require further investigation.

In conclusion, a total of 1,160 and 756 DEGs were identified in the pre-invasive and invasive stages of cervical cancer, respectively. The GO enrichment analysis demonstrated that the DEGs were primarily involved in the immune response and regulation of the cell cycle, in the pre-invasive and invasive stages, respectively. These findings indicate that the expression of key genes differs between the pre-invasive and invasive stages of cervical cancer progression. Based on the analysis of the regulatory networks, a total of 18 and 26 key miRNAs were screened in the pre-invasive and invasive stages, respectively. It is hypothesized that these miRNAs are involved in the malignant transformation of cervical cancer cells. In addition, these miRNAs may have a function as novel biomarkers in cervical cancer diagnosis and detection, and as therapeutic targets in this disease. Further studies in independent patient cohorts are required, in order to validate the potential roles of these miRNAs.

## Figures and Tables

**Table I:** Functional enrichment results of differentially expressed genes from NE samples and HSIL samples.

Category	ID	Description	FDR	Count	Gene
GOTERM_BP_FAT	GO:0002526	Acute inflammatory response	3.06E-04	23	CLU, SERPING1, APOL2…
GOTERM_CC_FAT	GO:0031975	Envelope	0.008979	69	HCCS, CYP24A1, S100A6…
GOTERM_CC_FAT	GO:0005783	Endoplasmic reticulum	0.009737	96	TUSC3, VAPB, LMAN2L…
GOTERM_BP_FAT	GO:0006959	Humoral immune response	0.014053	18	PSMB10, POU2AF1, ST6GAL1…
GOTERM_BP_FAT	GO:0006956	Complement activation	0.014251	13	C4A, C3, C4B…
GOTERM_BP_FAT	GO:0006259	DNA metabolic process	0.01695	59	XRCC4, CTCF, PTTG1…
GOTERM_BP_FAT	GO:0002541	Activation of plasma proteins involved in acute inflammatory response	0.018628	13	C4A, C3, CFB…
GOTERM_CC_FAT	GO:0031967	Organelle envelope	0.015595	68	HCCS, CYP24A1, S100A6…
GOTERM_CC_FAT	GO:0031090	Organelle membrane	0.021051	105	HCCS, CYP24A1, TUSC3…
GOTERM_CC_FAT	GO:0044432	Endoplasmic reticulum part	0.02565	44	ARL6IP1, DERL2, TUSC3…
GOTERM_BP_FAT	GO:0051605	Protein maturation by peptide bond cleavage	0.045871	18	C4A, CFB, C4B…
GOTERM_BP_FAT	GO:0016064	Immunoglobulin mediated immune response	0.046239	14	XRCC4, C4A, MSH2…
GOTERM_BP_FAT	GO:0019724	B cell mediated immunity	0.070007	14	XRCC4, C4A, MSH2…
GOTERM_CC_FAT	GO:0005740	Mitochondrial envelope	0.066884	49	HCCS, CYP24A1, COX5A…

NE, normal squamous cervical epithelium; HSIL, high-grade squamous intraepithelial cervical lesion; GO, Gene Ontology; FDR, false discovery rate; CLU clusterin; SEPRING1, human C1-inhibitor; APOL2, apolipoprotein L2; HCCS, cytochrome c-type heme lyase; TUSC3, tumor suppressor candidate 3; VAPB, vesicle-associated membrane protein-associated protein B/C; LAMN2L, lectin mannose-binding 2-like; PSMB10, protesome subunit β-type 10; ST6GAL, sialyltransferase; XRCC4, X-ray repair cross-complementing protein 4; CTCF, CCCTC-binding factor; CFB, complement factor B; ARL6IP1, ADP-ribosylation factor-like protein 6-interacting protein 1; DERL2, Derlin-2; MSH2, MutS protein homolog 2; COX5A, cytochrome *c* oxidase subunit VA.

**Table II:** Functional enrichment results of differentially expressed genes from invasive SCC of the cervix samples and HSIL samples.

Category	ID	Description	FDR	Count	Gene
GOTERM_CC_FAT	GO: 0031981	Nuclear lumen	7.07E-20	131	PNMA3, STK38, PKMYT1…
GOTERM_BP_FAT	GO: 0007049	Cell cycle	1.25E-19	98	TTK, AURKA, BRCA2…
GOTERM_CC_FAT	GO: 0070013	Intracellular organelle lumen	1.06E-17	145	CDKN2A, OIP5, SRRM2…
GOTERM_CC_FAT	GO: 0043233	Organelle lumen	1.20E-17	147	SRRM2, SMARCD1, SRRM1…
GOTERM_CC_FAT	GO: 0031974	Membrane-enclosed lumen	2.83E-17	148	TFDP2, POLQ, WDHD1…
GOTERM_BP_FAT	GO: 0022403	Cell cycle phase	4.80E-16	64	PSMC3IP, CDK10, TUBG1…
GOTERM_CC_FAT	GO: 0005654	Nucleoplasm	2.79E-15	90	MCM3, UBN1, WEE1…
GOTERM_BP_FAT	GO: 0000279	M phase	6.61E-15	55	CDCA3, STAG1, CDC6…
GOTERM_BP_FAT	GO: 0022402	Cell cycle process	7.36E-15	74	INHBA, REC8, PA2G4…
GOTERM_CC_FAT	GO: 0005694	Chromosome	4.77E-13	59	BLM, HIST1H2AG, NEK2…
GOTERM_CC_FAT	GO: 0043228	Non-membrane-bounded organelle	7.88E-13	173	KNTC1, TTK, AURKA…
GOTERM_CC_FAT	GO: 0043232	Intracellular non-membrane-bounded organelle	7.88E-13	173	RAD9A, ACTN3, MYH9…
GOTERM_BP_FAT	GO: 0000278	Mitotic cell cycle	6.72E-12	54	NCAPH, NCAPG, ZWILCH…
GOTERM_BP_FAT	GO: 0051301	Cell division	8.94E-11	46	ESPL1, MYH9, CDC25C…
GOTERM_BP_FAT	GO: 0048285	Organelle fission	1.25E-10	40	RAD21, NCAPG, OIP5…

SCC, squamous cell carcinoma; HSIL, high-grade squamous intraepithelial cervical lesion; GO, Gene Ontology; FDR, false discovery rate; PNMA3, paraneoplastic Ma antigen 3; STK34, serine threonine kinase 38; PKMYT1, protein kinase, membrane-associated tyrosine/threonine 1; AURKA, Aurora kinase A; CDkN2A, cyclin-dependent kinase inhibitor 2A; OIP5, opa interacting protein 5; SRRM1/2, serine/arginine repetitive matrix 1/2; SMARCD1, SW1/SNF-related matrix-associated actin-dependent regulator of chromatin subfamily D member 1; TFDP2, transcription factor Dp-2; WDHD1, WD repeat and HMG-box DNA binding protein 1; PSMC3IP, PSMCS interacting protein; CDK10, cyclin-dependent kinase 10; TUBG1, tubulin γ 1; MCM3, minichromasome maintenance complex component 3; UBN1, ubinuclein 1; CDCA3, cell division cycle associated 3; STAG1, stromal antigen 1; CDC6/25C, cell division cycle 6/25C; INHBA, inhibin βA; PA2G4, proliferation-associated 2G4; HIST1H2AG, histone cluster 1 H2ag; NEK2, NIMA-related kinase 2; KNTC1, kinetochore assoicated 1; RAD9A, RAD9 homolog A; MYH9, myosin heavy chain 9; NCAPH/G, non-SMC condensin 1 complex subunit H/G; ZWILCH, zwiltch kinetochore protein; ESPL1, extra spindle poles like 1; OIP5, opa-interacting protein 5.
